# Supplementary material for: Medical challenges and unmet needs of individuals with 22q11.2 deletion syndrome as perceived by caregivers: A thematic analysis and natural language processing-based thematic extraction
Source: PLOS Ment Health. 2026 Apr 3;3(4):e0000582. doi: 10.1371/journal.pmen.0000582 (PMC13048488; doi:10.1371/journal.pmen.0000582)
Supplement: S2 File — These examples are provided to illustrate the model’s internal reasoning process. (DOCX) [file pmen.0000582.s002.docx]

**Supplementary S2 file**

This file provides English translations of representative raw outputs generated by the NLP model during the thematic extraction process. The outputs illustrate how the model produced two independent analytic perspectives, labeled as “Researcher A” and “Researcher B,” which represent simulated analytic roles within the model rather than human researchers. These perspectives were subsequently compared by the model to derive thematic interpretations and reach internal agreement. Numerical identifiers in the outputs refer to response IDs that the model associated with each theme. However, because the NLP model showed limited performance in accurately classifying individual responses into specific themes, these response IDs could not be regarded as representative excerpts. For this reason, the response IDs were omitted from the presentation of the main results (Table 3).

Initial Code of Researcher A:

[Medical & Welfare] Comprehensive Support and Coordination: [3,6,8-9,14,17,20,25,27,30,32,34,36,38,40,42,44,46,48,50]

[Medical & Welfare] Specialized Knowledge and Explanation: [19-20,22,24,26,27,31,33,35,37,39,41,43,45,47,48,51,52,54,56]

[Medical & Welfare] Early Detection and Risk Management: [50-51,53]

[Education] Support Based on Children's Developmental Stages: [9,11,13,15,18,21,23,24,26,27,29,31,33,35,37-30,41,43,45]

Mental Care and Counseling: [1,4,5,10,12,16,28,36,49,55]

[Society & Community] Support for Reducing Parental Burden: [7,14-15,25,32,34,42,44,46,57]

Initial Code of Researcher B:

Consideration for Children's Health and Development: [9,10,18,21,23,24,26-27,29,31,33,35,37,39,41]

[Medical & Welfare] Necessity of a Comprehensive Support System: [3,6,8,10,12,14,17,20,22-23,25,27,30,32,34,36,38,40]

Mental Health and Psychological Support: [1,4-5,9,12,16,28,36,49,55]

[Society & Community] Addressing Parental Isolation: [7,11,13,15,25,32,42,44,46-47,57]

Theme Proposal and Adjustment:

Researcher A:

"I propose the overarching category of 'Medical and Welfare.' This relates not only to the need for specialized care and early intervention but also to coordination issues. It should be discussed within the broader framework of 'comprehensive improvements in medical and welfare services.'"

Researcher B:

"Indeed, these issues are interconnected. I propose three main themes: 'Children’s Health and Development,' 'Mental Health Support,' and 'Strengthening Parental Support Networks through Social and Community Collaboration.' However, an overarching framework for 'Proposals for Improving Medical and Welfare Services' is also necessary."

Final Response:

Child-Centered Approach:

Importance of care and support tailored to children's health and developmental stages [9,21]

Demand for mental health and psychological support [4-5,36], particularly considering the emotional well-being of both children and parents.

Comprehensive Improvement of Medical and Welfare Services:

Necessity of comprehensive medical consultation and coordination by specialists [17]

Risk management and early intervention from an early stage [48,50-51], with an emphasis on the presence of doctors knowledgeable about chromosomal abnormalities.

Parental Support Through Social and Community Networks:

Reducing isolation through information sharing and consultation services [7]

Educational support to deepen understanding of child care and development [15,29], including interactions with other parents.
