# Supplementary material for: Medical challenges and unmet needs of individuals with 22q11.2 deletion syndrome as perceived by caregivers: A thematic analysis and natural language processing-based thematic extraction
Source: PLOS Ment Health. 2026 Apr 3;3(4):e0000582. doi: 10.1371/journal.pmen.0000582 (PMC13048488; doi:10.1371/journal.pmen.0000582)
Supplement: S1 Table — (DOCX) [file pmen.0000582.s003.docx]

Table S1. Clinical characteristics of participants who did not provide free-text responses in the medical needs section (n = 60)

| Person with 22q11.2 deletion syndrome | | | | |  |
| --- | --- | --- | --- | --- | --- |
| Age, mean (sd) | |  |  | 11.5 | (7.1) |
| Women or girl, num (%) | | |  | 30 | (50.0) |
| Lifetime comorbidities, mean ( SD ) | | | | 4.0 | (1.7) |
| Lifetime comorbidities, N (%) | | |  |  |  |
|  | Congenital heart disease | | | 49 | (81.7) |
|  | Immune system disorder | | | 15 | (25.0) |
|  | Endocrine disorder | |  | 21 | (35.0) |
|  | Gastrointestinal disease | | | 14 | (23.3) |
|  | Otorhinolaryngology/maxillofacial disease | | | 44 | (73.3) |
|  | Orthopedic disease | | | 21 | (35.0) |
|  | Growth/developmental disorder | | | 54 | (90.0) |
|  | Psychiatric/neurological disorder | | | 11 | (18.3) |
|  | Other |  |  | 9 | (15.0) |
