# Supplementary material for: Medical challenges and unmet needs of individuals with 22q11.2 deletion syndrome as perceived by caregivers: A thematic analysis and natural language processing-based thematic extraction
Source: PLOS Ment Health. 2026 Apr 3;3(4):e0000582. doi: 10.1371/journal.pmen.0000582 (PMC13048488; doi:10.1371/journal.pmen.0000582)
Supplement: Attachment — Submitted filename: Response_to_Reviewers_260126.docx [file pmen.0000582.s005.docx]

Journal Requirements:

**1. Please provide additional details regarding participant consent. In the ethics statement in the Methods and online submission information, please ensure that you have specified (1) whether consent was informed and (2) what type you obtained (for instance, written or verbal, and if verbal, how it was documented and witnessed). If your study included minors, state whether you obtained consent from parents or guardians. If the need for consent was waived by the ethics committee, please include this information.**

**If you are reporting a retrospective study of medical records or archived samples, please ensure that you have discussed whether all data were fully anonymized before you accessed them and/or whether the IRB or ethics committee waived the requirement for informed consent. If patients provided informed written consent to have data from their medical records used in research, please include this information.**

***Response***

Thank you for this helpful comment. We have added detailed information regarding the consent procedure in the Ethical Considerations section of the Methods.

This study was conducted using a web-based survey. On the first page of the survey, participants were provided with written information about the study purpose, the voluntary nature of participation, the anonymity of data, and their right to withdraw. In accordance with the approval from the institutional ethics committee, informed consent was obtained through participants’ initiation of the survey—submission of responses was considered to indicate informed consent. All participants were adults and primary caregivers; therefore, no minors were included, and consent from parents or legal guardians was not required.

This study did not involve any retrospective review of medical records or the use of archived samples; it relied solely on data obtained through a web-based survey. All data were fully anonymized at the time they were accessed by the researchers. Therefore, issues regarding informed consent for the use of medical records or the waiver of such consent by the ethics committee were not applicable to this study.

The revised text has been added to the Methods section as follows.

***Deleted***: “Participants were instructed about the purpose of this study on the first page of the web-based survey. We also explained that informed consent was obtained through the participant's response to the survey.”

***Added***: “On the first page of the web-based survey, participants were provided with written information regarding the study purpose, the voluntary nature of participation, the anonymity of the data collected, and their right to withdraw. In accordance with the approval granted by the institutional ethics committee, informed consent was obtained through the participants’ initiation of the survey, whereby the submission of responses was considered to indicate informed consent. All participants were adults and primary caregivers; therefore, no minors were included, and parental or guardian consent was not required.”

**2. Please amend your detailed Financial Disclosure statement. This is published with the article. It must therefore be completed in full sentences and contain the exact wording you wish to be published.**

**i. State what role the funders took in the study. If the funders had no role in your study, please state: “The funders had no role in study design, data collection and analysis, decision to publish, or preparation of the manuscript.”**

***Response***

Thank you for pointing this out. We have revised the Financial Disclosure statement to ensure that it is written in complete sentences and includes the required information regarding the role of the funders. As none of the funders were involved in the study design, data collection and analysis, decision to publish, or preparation of the manuscript, we have included the standard wording recommended by the journal.

The revised Financial Disclosure statement now reads as follows:

“This study was partially supported by JSPS KAKENHI (Grant Numbers JP21H05171, JP21H05174, JP21H00451, JP21K13474, and JP23K27525), the Research and Development Grants for Comprehensive Research for Persons with Disabilities from the Japan Agency for Medical Research and Development (AMED) (Grant Number: 20ek0109369), the AMED Program for Research Ethics and Social Co-creation (Grant Number: JP25oa0439005h0001), the International Research Center for Neurointelligence (WPI-IRCN) at The University of Tokyo Institutes for Advanced Study (UTIAS), and the Yuumi Memorial Foundation for Home Health Care. The funders had no role in study design, data collection and analysis, decision to publish, or preparation of the manuscript.”

**3. Please ensure that your Ethics Statement is available in its entirety at the beginning of your Methods section, under a subheading 'Ethics Statement'.**

***Response***

We have revised the manuscript by replacing the subheading “Ethical considerations” with “Ethics Statement” and ensured that the complete ethics information appears at the beginning of the Methods section as requested.

**4. We have noticed that you have cited Table 2 in the manuscript file but there are no corresponding tables in the manuscript. Please amend your manuscript to include this table, noting that tables should not be uploaded as individual files.**

***Response***

Thank you for pointing this out. The table cited as Table 2 in the manuscript did not display because a table number and title had not been assigned. Because we have added Table 1 (demographic), the numbering of the subsequent tables has shifted. We have corrected this issue in the revised manuscript by adding the table as “Table *3*. Categories extracted using natural language processing” and ensuring that it is properly inserted within the manuscript.

**5. We have noticed that you have uploaded Supporting Information files, but you have not included a list of legends. Please add a full list of legends for your Supporting Information files after the references list.**

***Response***

Thank you for pointing this out. We have now added a complete list of legends for all Supporting Information files after the references section. The Supporting Information includes the English translations of the survey prompts originally written in Japanese, which could not be fully included in the main text.

The revised legend has been *added* as follows:

S1 File. English translations and materials used for prompt engineering procedures.

This file includes the English translations of the step-by-step prompts, mock outputs, and revised prompts used in the prompt engineering procedures applied in this study.

**6. We note that you have indicated that there are restrictions to data sharing for this study. For studies involving human research participant data or other sensitive data, we encourage authors to share de-identified or anonymized data. However, when data cannot be publicly shared for ethical reasons, we allow authors to make their data sets available upon request. For information on unacceptable data access restrictions, please see http://journals.plos.org/plosone/s/data-availability#loc-unacceptable-data-access-restrictions.**

**a) If there are ethical or legal restrictions on sharing a de-identified data set, please explain them in detail (e.g., data contain potentially identifying or sensitive patient information, data are owned by a third-party organization, etc.) and who has imposed them (e.g., a Research Ethics Committee or Institutional Review Board, etc.). Please also provide contact information for a data access committee, ethics committee, or other institutional body to which data requests may be sent.**

**b) If there are no restrictions, please upload the minimal anonymized data set necessary to replicate your study findings to a stable, public repository and provide us with the relevant URLs, DOIs, or accession numbers. Please see http://www.bmj.com/content/340/bmj.c181.long for guidelines on how to de-identify and prepare clinical data for publication. For a list of recommended repositories, please see https://journals.plos.org/plosone/s/recommended-repositories. You also have the option of uploading the data as Supporting Information files, but we would recommend depositing data directly to a data repository if possible.**

**Please update your Data Availability statement in the submission form accordingly.**

***Response***

Thank you for your detailed comments regarding data sharing. The dataset generated in this study includes free-text responses, and complete removal of potentially identifiable information cannot be fully guaranteed. Therefore, there are ethical restrictions on publicly sharing the dataset. These restrictions have been imposed by the Ethical Committee, Faculty of Medicine, The University of Tokyo.

Although the full dataset cannot be made publicly available, we are able to provide a minimal anonymized dataset upon reasonable request, in accordance with the ethical guidelines. Data requests may be sent to the 22q Research Office at the University of Tokyo (22q.research@gmail.com), which serves as the designated contact point for data access inquiries.

We have updated the Data Availability Statement in the submission form accordingly to reflect this information.

**Reviewer #1: To Authors:**

**The manuscript addresses an important and underexplored topic concerning the medical and psychosocial needs of individuals with 22q11.2 deletion syndrome (22q11DS) and their caregivers. The dual application of traditional thematic analysis (TA) and natural language processing (NLP)-based thematic extraction is timely and potentially valuable.**

**Comment 1: The research aim and rationale require clearer logical coherence. Specifically, the manuscript should more explicitly articulate how the NLP-based analysis advances existing qualitative approaches, beyond efficiency and scalability. A sharper statement of the theoretical and methodological gap that the study fills would strengthen the Introduction.**

***Response***

Thank you for this comment. We agree that the Introduction did not sufficiently articulate how the NLP-based analysis contributes to qualitative research beyond improvements in efficiency and scalability, nor did it clearly describe the theoretical and methodological rationale underlying our approach.

In response, we have revised the Introduction to clarify the following points.

*Added*: We adopted a critical realist perspective, which assumes that complex phenomena cannot be fully captured through a single analytic method and are better understood by integrating multiple methodological lenses. Accordingly, we implemented method triangulation by combining human-led thematic analysis with NLP-based automated category extraction. This approach allows us to complement potential heuristics and biases that may arise during researcher-driven coding, while providing a more comprehensive and structured perspective on the diverse and complex needs characteristic of individuals with 22q11.2 deletion syndrome. These additions strengthen the theoretical coherence of the study and articulate the methodological gap that the combined qualitative–computational approach aims to address.

**Comment 2: The methodological rigor and transparency are generally adequate, but several details need clarification for reproducibility. The authors should describe the exact prompts used for the NLP analysis (or summarize them in the main text rather than relegating all to the supplement) and specify how the “themes” were algorithmically defined and aggregated. For the manual TA, the coding framework and the process for category refinement should be explained more concretely, including how discrepancies were resolved and how reflexivity was maintained among coders.**

***Response***
Thank you for your comment. We agree that the description of the prompts used in the NLP analysis was limited, focusing primarily on the names of the techniques rather than the actual procedural instructions given to the model. In the revised manuscript, we have therefore added a clearer explanation of the structure and purpose of the prompts.

Specifically, we have clarified that the NLP workflow was driven entirely by prompt engineering rather than by external algorithms such as embeddings or clustering. The prompts were designed to function as a step-by-step algorithm for thematic extraction, guiding the model through processes analogous to manual thematic analysis. The main components of the prompts included: (1) a system prompt instructing the model to act as a qualitative research scholar, (2) stepwise instructions that directed the model to conduct familiarization, generate initial codes, compare and integrate codes, and derive final themes, (3) a tree-of-thoughts–style structure simulating two researchers (A and B) who independently propose and compare interpretations, enabling a consensus-building process, and (4) iterative refinement using mock outputs. These additions clarify how themes were algorithmically defined and consolidated within the NLP analysis.

As the full prompts are lengthy and contain detailed stepwise instructions as well as mock outputs, including them in the main text would reduce readability. For this reason, the complete prompts have been placed in the Supplementary Information (S1 File). These revisions improve transparency and reproducibility of the NLP-based thematic extraction.

***Deleted*** (in Methods)*:*
Thematic extraction was conducted by inputting all free-text responses into the model, along with detailed descriptions of the TA methodology. This process was carried out without reference to the results of the manual TA.

Prompt engineering techniques were applied to enhance task performance. The following three techniques were employed in this study (16) :

Zero-shot chain of thought: Detailed step-by-step task descriptions were included in the input text.

Mock: The task process was followed by the phrase "Did you understand?" to ensure the model comprehended the task before proceeding.

Tree of thoughts: A method where multiple thought sequences are explored through branching, implemented via prompt engineering. Example: "Imagine that multiple experts are responding to this question." "Each expert writes down one step of their thought process and shares it with the group. Then, all experts proceed to the next step, and so on." "If an expert identifies an error at any point, they exit the process."

The step-by-step prompt, the corresponding mock output, and the revised prompt based on that output will be included in the supplementary materials.

***Added*** (in Methods): In the NLP-based thematic extraction, we did not employ external algorithms such as embeddings or clustering. Instead, we used a prompt-engineering approach in which the model followed a stepwise set of analytic procedures. This process was carried out without reference to the results of the manual TA. A system prompt instructed the model to act as a qualitative research scholar, and subsequent prompts directed it through sequential stages analogous to manual thematic analysis, including familiarization with the data, generation of initial codes, comparison and integration of related codes, and the derivation and naming of themes. The prompts also incorporated a tree-of-thoughts structure in which two simulated researchers (A and B) independently proposed interpretations and reached consensus through comparison, as well as iterative refinement using mock outputs. This structured prompting allowed the model to execute analytic steps corresponding to the hierarchical stages of manual thematic analysis (initial codes → categories → themes). Because the full prompts are lengthy and include detailed stepwise instructions, they are provided in the Supplementary Information (S1 File).

We also agree that the manuscript did not provide sufficient detail regarding the construction of the coding framework, the refinement process, the resolution of discrepancies between coders, and the procedures used to maintain reflexivity. To improve methodological transparency, we have revised the Methods section to include the following details:

***Deleted*** (in Methods):
To ensure objectivity, the following steps were added:

1. Two researchers independently coded responses from a randomly selected subset of 16 participants (25%).

2. After confirming an inter-coder agreement rate of over 90%, the first author coded the remaining responses.

3. Categories were classified and named, with all categories reviewed among researchers.

***Added*** (in Methods):

To enhance rigor, two researchers independently generated initial codes from a randomly selected subset of 25% of the responses. Their coding notes were then compared, and similar codes were merged and organized into a hierarchical structure (initial codes, subcategories, categories). The definitions of individual codes were iteratively refined to reduce redundancy and ambiguity, resulting in clear operational definitions. Relationships among subcategories were examined to structure them toward higher-order categories.

After independent coding, discrepancies between the two coders were identified and discussed in depth, and consensus was reached through iterative discussion. The intercoder agreement rate (90%) was calculated using simple percentage agreement. Once consensus on the coding framework was established, the first author proceeded to code the remaining data.

Reflexivity was maintained throughout the analysis. The two coders brought distinct forms of expertise and positionality: the first author is a psychiatrist working in a specialized outpatient clinic for individuals with 22q11.2 deletion syndrome and is a beginner in thematic analysis, whereas the second author is a clinical psychologist with substantial qualitative research experience and close engagement with participants through recruitment, communication, and follow-up. Analytic memos were shared to reflect on how these backgrounds might influence interpretations, and potential biases were discussed and adjusted during the refinement of categories and themes. This iterative process ensured that interpretations did not become overly shaped by a single researcher’s standpoint.

**Comment 3: While the use of an open-source LLM in a secure offline environment is commendable, the technical explanation could be made more accessible to readers in health sciences. Briefly explain what distinguishes the “quantized” model from the original and why this configuration was chosen. Readers unfamiliar with machine learning may find it difficult to follow the computational logic without some interpretive guidance.**

***Response***

Thank you for this comment. We agree that the explanation of the “quantized” model could be made more accessible to readers in the health sciences. We have revised the manuscript to clarify the following points in the Methods section.

***Added*** (in Methods):

To protect personal information, all analyses were conducted in a fully offline environment. Inference was performed using two NVIDIA RTX 6000 Ada GPUs (48 GB each). We selected a quantized model that could be loaded entirely into the available GPU memory to enable secure, local execution. Quantization refers to reducing the numerical precision of model weights (e.g., from 16-bit to 4- or 8-bit), which substantially lowers memory and computational requirements while maintaining sufficient performance for tasks such as thematic extraction. We used a quantized version of the open-source large language model CohereForAI/c4ai-command-r-plus (14), specifically bartowski/c4ai-command-r-plus-08-2024-GGUF (Q6_K) (15), which provided the highest precision compatible with our hardware constraints. Q6_K corresponds to a 6-bit quantization scheme in the GGUF format. No additional training or domain-specific fine-tuning was performed.

***Deleted*** (in Methods): To protect personal information, all analyses were conducted offline. Inference was conducted using two NVIDIA RTX 6000 Ada graphics processing units (GPUs) with 48 GB of memory per unit, using a quantised model that fit entirely within the GPU memory. We used a quantized version of the open-source large language model (CohereForAI/c4ai-command-r-plus (14), quantized as bartowski/c4ai-command-r-plus-08-2024-GGUF(Q6_K) (15)). We did not perform any additional training on medical knowledge or fine-tuning for the medical domain.

**Comment 4: The comparative validity between the manual and NLP-based analyses is discussed qualitatively but would benefit from quantitative or structured evaluation. Even if statistical concordance is not possible, presenting an explicit mapping table showing which categories overlapped, diverged, or were newly generated (with frequency counts or exemplar quotations) would enhance credibility and reproducibility.**

***Response***

Thank you for this insightful comment. As noted, the open-source LLM used in this study (NLP-based thematic extraction) exhibited limitations in its ability to reliably assign individual free-text responses to specific subcategories. Due to model characteristics and constraints in inference accuracy, many responses were disproportionately assigned to a single subcategory (e.g., Child-centered Approach 1a). As a result, calculating subcategory-level frequencies or treating the automatically generated quotations as “representative exemplars” would not have been methodologically appropriate.

For this reason, we treated the quotations generated by the NLP model as supplementary outputs only.

To ensure that each subcategory was represented accurately, all exemplar quotations used in the revised manuscript were selected directly by the researchers based on the original free-text data obtained through manual thematic analysis, rather than relying on model-generated excerpts.

Furthermore, the comparison between manual TA and the NLP-based analysis was conducted not at the level of reassigning each individual response to NLP-derived subcategories, but at the conceptual, theme-level, based on the meanings indicated by the category and subcategory labels extracted by each method.

To enhance transparency, we created a mapping table that clearly indicates which themes overlapped across the two approaches and which were unique to either the manual TA or the NLP-based extraction. This table has been added to the revised manuscript.

We believe that this structured approach allows us to illustrate the similarities and differences between the two analytic methods without overinterpreting the precision of model-based classifications, thereby providing an appropriate assessment of their comparative validity. We have also clarified in the Limitations section that subcategory-level automatic classification and quantitative concordance analyses remain areas for future methodological improvement.

We hope that these revisions improve the clarity, transparency, and reliability of the comparison between manual TA and NLP-based thematic extraction.

In Results, we *removed* the “Excerpt from narrative” column from the NLP-based thematic extraction results (in Table 3), as these excerpts were not used in the final comparison.

In Discussion, we have *added* Table 5 as the mapping table.

We also ***added*** the following paragraph in the beginning of Discussion:

We interpret the findings by comparing the results of the manual thematic analysis and the NLP-based thematic extraction. We created a mapping table that summarizes the overlaps and divergences between subcategories. The differences observed between the two approaches reflect their distinct analytic characteristics. Manual TA enabled the generation of themes informed by researchers’ clinical experience and interpretive judgment, resulting in themes that were identified only through human analysis. Conversely, the NLP approach identified themes that were less salient from a human interpretive perspective, thereby complementing the manual analysis.

Upon review, we found omissions in the mapping of subcategory correspondences. We have corrected these issues and revised the relevant sections as follows.

***Deleted***: Category 1 identified via manual TA; Subcategory 2a identified via NLP

***Added***: Subcategory 1a and 1b identified via manual TA; Subcategory 2a and 2b identified via NLP

**Comment 5: The Results section is well organized, but the tables could be improved by integrating key quotes from caregivers that exemplify the derived subcategories. Doing so would make the findings more vivid and support claims about interpretive alignment between the two methods.**

***Response***

Thank you very much for this constructive suggestion. We agree that integrating exemplar quotations that represent the derived subcategories can make the findings more vivid and further support the interpretive alignment between the manual and NLP-based analyses.

As noted in our response to the previous comment, we have revised the manuscript by adding exemplar quotations to the mapping table for the subcategories that were commonly identified by both the manual thematic analysis (TA) and the NLP-based thematic extraction. These quotations help illustrate the thematic content in a concrete and reader-friendly manner.

However, due to limitations in the inference accuracy of the LLM used in this study, the NLP model tended to assign a disproportionately large number of responses to a single subcategory, making it inappropriate to treat the model-generated quotations as representative examples of each NLP-derived subcategory. For this reason, all exemplar quotations included in the revised manuscript were selected manually from the original free-text responses based on the manual TA.

**Comment 6: In the Discussion, the integration of findings with prior literature is appropriate, yet parts of the argument overstate the implications. The claim that “NLP could be useful to analyze large-scale free-text data on medical needs” should be qualified as preliminary given the limited sample size (n = 65 free-text responses). The authors might more cautiously frame NLP as a complementary exploratory tool rather than a replacement for manual analysis.**

***Response***

Thank you for this important comment. As you noted, our dataset consisted of 65 free-text responses, and therefore the sample size was limited. We agree that the statement suggesting that “NLP could be useful for analyzing large-scale free-text data on medical needs” may have conveyed an overly generalized implication in light of this limitation.

We have revised the statements regarding the potential usefulness of NLP to adopt a more cautious and tentative tone, explicitly describing our findings as preliminary. In the revised manuscript, we clarify that NLP should not be understood as a replacement for manual thematic analysis, but rather as a complementary and exploratory tool that can offer additional perspectives and suggest potential patterns. Furthermore, the discussion of its applicability to large-scale free-text data has been rephrased to emphasize that this possibility represents only a tentative implication based on the characteristics observed in our dataset.

We have revised the Abstract as follows.

***Deleted***:

This study implies that NLP could be useful to analyze large-scale free-text data on medical needs, providing valuable insights for healthcare policy considerations.

***Added***:

Given the limited size of our dataset, the implications of this study should be regarded as preliminary. Nevertheless, our findings suggest that NLP may serve as a useful exploratory approach to complement manual TA when analyzing larger free-text datasets on medical needs in future research. NLP should not be viewed as a replacement for manual TA, but rather as a supportive method that offers additional perspectives and potential patterns.

**Comment 7: The Limitations section is appreciated, but additional methodological caveats should be acknowledged—particularly the potential influence of prompt design on NLP outputs and the cultural context of Japanese caregiver narratives, which may constrain generalizability to other languages or health systems.**

***Response***

Thank you for pointing out the additional methodological considerations that should be addressed in the Limitations section.

The potential influence of prompt design on NLP outputs is an important issue. We have added a statement acknowledging that the prompts used in this study may have affected the thematic extraction process, and that the structure and instructions within the prompt could shape the model’s response patterns. We also clarified that the results produced by the NLP model depend on the specific prompt configuration employed, and that future research should examine how different prompts or model settings may alter the outcomes.

Regarding the fact that our data are based on narratives from caregivers in Japan, we added a discussion acknowledging that cultural and institutional contexts may influence both the style of narration and the expression of needs. Because the responses likely reflect features of the Japanese healthcare system, family dynamics, and social context of caregiving, we clarified that generalizability to other languages or healthcare systems may be limited and requires caution.

We ***added*** the following sentences in the Limitation section.

Fourth, NLP outputs may be substantially influenced by prompt design. The prompts used in this study could have shaped the thematic extraction process and the resulting category structure, and different prompts or model configurations may yield different results. Future research should compare multiple prompt and model conditions to examine the robustness of NLP-derived findings.

Fifth, the free-text responses analyzed in this study were generated by caregivers in Japan, and thus may reflect cultural and institutional factors that shape narrative style and the expression of needs. Given the influence of the Japanese healthcare system, family norms, and caregiving context, generalizability to other languages or healthcare systems should be approached with caution.

**Comment 8: Regarding novelty and contribution, the study’s strength lies in demonstrating the feasibility of privacy-preserving NLP-assisted qualitative analysis within medical research. To make this contribution more visible, the authors could reorganize the Discussion around this methodological innovation and discuss its potential integration into participatory or mixed-methods research frameworks.**

***Response***

Thank you for this valuable comment. We agree that the novelty and contribution of our study—demonstrating the feasibility of conducting NLP-assisted qualitative analysis in a privacy-preserving manner within medical research—should be articulated more explicitly.

In response to your suggestion, we reorganized the beginning of the Discussion to foreground the significance of the offline, privacy-preserving NLP workflow established in this study. We also revised the text to more clearly discuss how this methodological approach could be integrated into participatory research frameworks.

The Discussion has been modified as follows.

***Deleted*** (previous final paragraph):

In a previous study which applied ChatGPT-based TA, they used non-open-source large language models (LLMs), requiring input data to be uploaded to the cloud. This posed a limitation for analyzing sensitive qualitative data involving privacy (22). By contrast, we employed an open-source LLM locally, allowing us to process privacy-related qualitative information securely and offline. We established an offline framework for fully automated theme extraction and naming, which may assist researchers in gaining an overview of the key characteristics of large-scale qualitative data before conducting a full manual review (10). In future large-scale studies involving qualitative data, NLP-based tools may serve as effective thematic extraction methods.

***Added*** (new opening paragraph):

A key methodological contribution of this study lies in demonstrating the feasibility of conducting NLP-assisted qualitative analysis within a fully offline, privacy-preserving environment. Previous applications of large language models (LLMs) for thematic analysis relied on cloud-based systems (10) (11), which posed challenges when handling sensitive personal narratives (22). In this study, we developed an analysis workflow that employs an open-source LLM executed entirely on local hardware, enabling secure processing of confidential qualitative data. This approach provides a practical framework for researchers working with protected health information while also possessing characteristics that make it potentially applicable to participatory research. Recent work has shown that NLP can extract major themes from large volumes of free-text data and complement traditional qualitative approaches (Bennis and Mouwafaq 2025) . Studies integrating NLP with thematic analysis further suggest that preliminary computational themes can serve as a useful starting point for collaborative interpretation (Di Basilio et al. 2024), indicating that NLP may support co-analysis in participatory research settings. Moreover, participant trust, psychological safety, and robust data protection are recognized prerequisites for the use of digital technologies in healthcare (Catapan et al. 2025) (Catapan et al., 2025), and secure, locally executed workflows can help create conditions in which participants feel comfortable sharing their experiences. Taken together, our workflow—which combines secure data handling with NLP-assisted thematic exploration—may provide a methodological foundation that supports participatory research involving collaborative analysis among patients, caregivers, and healthcare professionals.

**Reviewer #2: Comments to Authors**

**Sawai et al. reported the results of a qualitative study on the medical issues and unmet needs of individuals of 22q11.2 deletion syndrome in Japan. The study used data from a web-based survey and conducted a thematic analysis using manual coding and NLP methods. The authors highlighted the importance of comprehensive improvements in the healthcare system and support for caregivers. The authors also developed an automated thematic extraction workflow using NLP for better analysis of larger-scale qualitative data.**

**This study will contribute to clarifying the medical support needed by individuals with multiple conditions and their caregivers. However, there are several points that need to be addressed to further improve the manuscript.**

**Title**

**1. Since this study is based on free-text comments provided by caregivers, the authors need to consider which wording of the title is appropriate to describe this study; Medical challenges and unmet needs of individuals themselves or caregivers.**

***Response***

Thank you very much for this valuable comment. As the free-text responses analyzed in this study were written by caregivers (primarily parents), the medical challenges and unmet needs described in the dataset reflect both (1) issues experienced by individuals with 22q11.2 deletion syndrome as perceived by caregivers, and (2) caregivers’ own challenges and needs related to supporting their children.

We fully agree that the title should clearly communicate whose challenges and unmet needs are being examined. In response to this suggestion, we carefully reconsidered the wording of the title to ensure that it accurately represents the perspective captured in our data.

As a result, we have revised the title as follows to make the caregiver-based perspective explicit:

***Revised title***: “Medical challenges and unmet needs of individuals with 22q11.2 deletion syndrome ***as perceived by caregivers***: A thematic analysis and NLP-based thematic extraction.”

**Methods**

**2. The demographics of the participants is not listed in the manuscript. Although the demographics of the original 125 participants of the survey can be found in their previous papers, cited as ref. 6 and 12, the demographics of the 65 participants provided free-text responses should be presented.**

***Response***

Thank you for this helpful comment. We agree that presenting the demographic characteristics of the 65 participants who provided free-text responses is important for contextualizing the findings.

Although the demographics of the full survey sample (N = 125) are reported in our previous publications (refs. 6 and 12), we have now added the demographic information of the 65 participants as a new Table 1 in the revised manuscript. This table includes age, sex, relationship to the individual, and other relevant characteristics.

As a result of adding this new table, the numbering of the subsequent tables has shifted. We have updated all table numbers and corresponding citations in the manuscript accordingly.

We have ***added*** the following sentence to the Target Population subsection of the Methods:

“Descriptive statistics of respondent characteristics are shown in Table 1 (n = 65).”

**3. The authors should describe the procedure used to extract comments in this study. For example, how many of the 125 participants provided free-text comments, and among them, how many commented specifically on medical issues? In addition, were there any notable characteristics or trends between those who provided free-text comments and those who did not? If there were differences between 2 groups in factors such as the severity of the physical or mental condition of individuals or the participants’ regions of residence (e.g. urban and rural areas regarding medical access), the authors should discuss them. There may be distinctive motivations that encourage caregivers to leave comments.**

***Response***

Thank you for this comment. A total of 125 participants completed the survey, which included several free-text questions. Of these, 65 participants provided responses to the free-text question specifically addressing medical needs. In this study, we analyzed the responses from these 65 participants who provided substantive comments in the medical needs section.

To examine potential differences between participants who provided free-text responses (respondents) and those who did not (non-respondents), we present the characteristics of respondents in Table 1 and the characteristics of non-respondents in Supplementary Table S1. The comparison showed no substantial differences in mean age or sex distribution between the two groups. In contrast, respondents exhibited a greater variety of physical comorbidities, with slightly higher prevalence of conditions such as congenital heart disease, immune system disorders, and endocrine disorders. In addition, the prevalence of psychiatric disorders was approximately twice as high among respondents compared with non-respondents.

These patterns suggest that families experiencing more complex physical and mental health challenges may have been more motivated to provide free-text comments on medical needs. This potential response bias indicates that the findings of this study may more strongly reflect the perspectives of individuals with relatively higher healthcare needs, a point that should be considered when interpreting the results. Accordingly, we have added this discussion to the Limitations section.

With respect to region of residence, information was collected only at the prefectural level, making it difficult to distinguish precisely between urban and rural areas. However, the proportion of participants residing in prefectures that include major metropolitan areas (e.g., Tokyo and Osaka) did not differ substantially between respondents and non-respondents.

***Added*** (in Limitation):

There also may be sampling bias between respondents (Table 1) and non-respondents (Table S1) to the free-text question on medical needs. Although no substantial differences were observed in mean age or sex distribution, respondents exhibited a greater variety of physical comorbidities, with slightly higher prevalence of conditions such as congenital heart disease, immune system disorders, and endocrine disorders. In addition, the prevalence of psychiatric disorders among respondents was approximately twice that observed among non-respondents (36.9% vs. 18.3%). These patterns suggest that families experiencing more complex medical and psychiatric challenges may have been more likely to provide free-text responses, and thus the results of this study may disproportionately reflect the perspectives of individuals with more complex healthcare needs.

**4. Regarding manual thematic analysis, the authors described “1. Two researchers independently coded responses from a randomly 89 selected subset of 16 participants (25%). 2. After confirming an inter-coder agreement rate of over 90%, the first author coded the remaining responses.”**

**Please specify whether the two researchers who manually coded the text in this study included the first author or were two independent individuals. Also, it is better to describe briefly whether there were any differences in their experience or expertise related to thematic analysis.**

***Response***

Thank you for this important comment. As suggested, we have clarified in the manuscript who conducted the manual thematic analysis and how their backgrounds differed.

In this study, the initial coding of the free-text responses was conducted independently by two researchers: the first author and the second author. The first author is a psychiatrist and was a beginner in thematic analysis; before starting the coding work, the first author received training and guidance from the second author. In contrast, the second author is a clinical psychologist with substantial experience in qualitative research and thematic analysis, and has been deeply involved in participant recruitment, communication, and follow-up throughout the study.

We have added this information to the Methods section (manual thematic analysis) so that readers can clearly understand who performed the coding and how their levels of experience and professional backgrounds differed.

We have added the following description to the manuscript accordingly.

***Added***:

Reflexivity was maintained throughout the analysis. The two coders brought distinct forms of expertise and positionality: the first author is a psychiatrist working in a specialized outpatient clinic for individuals with 22q11.2 deletion syndrome and is a beginner in thematic analysis, whereas the second author is a clinical psychologist with substantial qualitative research experience and close engagement with participants through recruitment, communication, and follow-up. Analytic memos were shared to reflect on how these backgrounds might influence interpretations, and potential biases were discussed and adjusted during the refinement of categories and themes. This iterative process ensured that interpretations did not become overly shaped by a single researcher’s standpoint.

**Results**

**5. The aim of this study appears to be to identify medical issues. Actually, in their previous work cited as ref. 6, the authors also divided discussions into medical, welfare, and educational domains. However, in the present results, particularly presented in table 2 (provided via NLP), it seems that contents related to the “welfare” domain are included. For example, category 2 contains the term “welfare services,” and category 3 includes “Social and Community Networks.”**

**The authors should clarify whether the target of this study is “medical” issues only, or both “medical and welfare” issues. Alternatively, if this overlap is due to technical limitations or characteristics of the NLP program they developed, it should be discussed.**

***Response***

Thank you very much for this insightful comment. We agree that it is important to clarify the scope of the domains addressed in this study, and we have revised the manuscript accordingly.

The questionnaire used in this study provided separate free-text fields for expressing needs in three domains: medical care, welfare, and education. In the present analysis, we included only the free-text responses written in the “medical care” section.

However, upon reviewing the actual responses, we found that caregivers often did not clearly distinguish between medical issues and welfare-related support needs. As a result, some statements in the medical section also described needs typically associated with the welfare domain. For example, one caregiver wrote:

“It would be helpful to have a centralized consultation point like a general medicine department and a coordinator such as a care manager or social worker.”

This illustrates that medical and welfare needs are closely intertwined in caregivers’ daily experiences, making them difficult to separate conceptually.

Because our NLP-based thematic extraction operates directly on the vocabulary and contextual information contained in the responses, it may identify themes that include welfare-related content when such content is present in the text. The appearance of welfare-related themes in Table 2 therefore reflects the nature of the responses themselves, rather than a technical limitation of the NLP approach.

We have added an explanation of these points to the Discussion section.

***Added***:

Although this study analyzed only the free-text responses written in the “medical care” section of the questionnaire, some caregivers described issues that spanned both medical and welfare domains. In practice, medical challenges and welfare support needs are often closely intertwined in the daily lives of families, making them difficult to distinguish (e.g. "It would be helpful to have a centralized consultation point like a general medicine department and a coordinator such as a care manager or social worker."). Consequently, some responses in the medical section included welfare-related content, such as the need for a centralized consultation point or coordination by a care manager or social worker. Because the NLP-based thematic extraction reflects the vocabulary and contextual information provided by respondents, welfare-related elements were also captured as themes. This overlap should therefore be understood as a characteristic of the responses themselves rather than a limitation of the NLP approach.

**6. Another concern regarding table 2 is that no excerpt is provided in cell 2a. Is there any reason to left this cell blank?**

***Response***

Thank you for pointing this out. We appreciate the opportunity to clarify why no excerpt was provided in cell 2a of Table 2 (Table 3 in the current manuscript), and we have revised the manuscript accordingly.

Although the NLP model used in this study produced reasonable results for theme extraction, it was not sufficiently reliable in assigning individual free-text responses to specific themes. While the model generated example segments that could be associated with some themes, we determined that it would be inappropriate to present these as representative excerpts of the themes without manual verification.

For this reason, we decided that excerpts should not be included for any of the NLP-derived themes, not only for cell 2a. We therefore revised Table 2 (Table 3 in the current manuscript) to maintain consistency by omitting excerpts across all NLP-generated categories.

These considerations have now been explained in the manuscript, and the table has been updated accordingly.

**Discussion**

**7. As above mentioned in the comment regarding the title, it is not clearly stated in the text whether the main focus of this study is on the challenges and unmet needs of the “individuals themselves” or those of the “caregivers”. This distinction could affect the content and interpretation in the Discussion.**

***Response***

Thank you for this comment. We agree that the manuscript did not sufficiently clarify whether the focus of this study lies on the challenges and unmet needs of the “individuals themselves” or those of the “caregivers.” We have now clarified this point in the revised manuscript.

This study examines medical challenges and unmet needs as described from the caregivers’ perspective. The free-text responses include both

(1) needs that caregivers perceive as necessary for the individuals themselves, and

(2) needs that caregivers personally experience in the course of accompanying medical visits or providing daily support.

Thus, the target of our analysis is not the individuals’ needs per se, but rather the set of needs and challenges as recognized and reported by caregivers.

We have added an explanation to the Discussion section to make it clear that the primary focus of this study is based on caregivers’ perspectives.

***Added***:

Because all free-text responses analyzed in this study were written by caregivers, the findings reflect caregivers’ perceptions of medical challenges and unmet needs rather than direct reports from individuals with 22q11.2DS themselves. These descriptions include both the needs that caregivers perceive as necessary for the individuals and the needs they personally experience in the course of accompanying medical visits or providing daily support. This distinction is important for interpreting the results, as the themes represent caregiver-recognized challenges and unmet needs, which may not fully align with the individuals’ own perspectives.

**8. The lack of a clear rationale for using two methods in this study makes the discussion ambiguous. It is unclear whether the main purpose of this study is (1) to understand broader issues using two methods, (2) to examine the consistency between the two methods, or (3) to demonstrate the significance of the NLP approach.**

**In the Introduction, the authors state, “Two studies applied ChatGPT-based TA, indicating that the manual TA and NLP-based thematic extraction were largely consistent (10) (11).” If the purpose corresponds to either (2) or (3) above, it is necessary to discuss the differences between the two methods in the current results.**

***Response***

Thank you for this important comment. We agree that the rationale for using two analytic methods was not sufficiently clear in the original manuscript. The primary purpose was (1) to understand broader issues using two methods.

Based on a critical realist perspective, we assumed that complex medical needs cannot be fully captured through a single analytic method. Therefore, we used NLP in a complementary manner to manual thematic analysis, particularly to illuminate themes that might not be fully identified through human-led coding alone, thereby enhancing the overall comprehensiveness of the analysis.

We have added clarification in the revised version.

***Added***:

In line with a critical realist perspective, we assumed that the complex medical needs of individuals with 22q11.2DS could not be fully captured through a single analytic method. Therefore, we employed manual thematic analysis and NLP-based thematic extraction in a complementary manner. By using NLP to illuminate themes that might not be fully identified through manual analysis alone, our aim was to obtain a more comprehensive and expansive understanding of the challenges and unmet needs described by caregivers.

**Reviewer #3: Overall Evaluation**

**This study explores medical and psychosocial needs of caregivers of individuals with 22q11.2 deletion syndrome (22q11DS) using both manual thematic analysis (TA) and natural language processing (NLP)-based thematic extraction with an open-source large language model (LLM). The topic is highly relevant, the dataset is ethically collected, and the attempt to apply offline NLP to sensitive qualitative data is novel and valuable.**

**However, across the Introduction, Methods, Results, Discussion, and Limitations, the paper lacks methodological transparency and conceptual coherence between manual and NLP analyses. The main concerns relate to (1) the unclear positioning of NLP within the study’s aims, (2) the absence of quantitative or procedural validation for AI-derived results, and (3) over-generalization of AI outputs without critical reflection on model behavior.**

**Below, comments are organized by section, with major and minor issues clearly indicated.**

**Major Points**

**1. Clarity of Study Aim and Role of NLP**

**(Section: Introduction, last two sentences)**

**The introduction does not clearly articulate whether the study aims to validate NLP as a replacement for manual TA, or to demonstrate feasibility of automatic extraction.**

**Clarify if NLP serves as (a) a methodological validation exercise, (b) a complement to human TA, or (c) an exploratory tool for scalability.**

**State explicitly how this objective shapes the study design and evaluation.**

***Response***

Thank you for this comment. As you pointed out, the original Introduction did not sufficiently clarify the role of NLP in this study. Our intention was not to validate NLP as a replacement for manual thematic analysis, but rather to use NLP as an exploratory tool that complements manual TA and supports a broader and more comprehensive understanding of the complex medical needs described by caregivers.

Grounded in a critical realist perspective, which assumes that complex phenomena cannot be fully captured through a single analytic method, we combined manual TA with NLP to leverage the strengths of both approaches. The rationale was that NLP could illuminate themes that might not be fully identified through manual analysis alone, thereby enhancing the overall comprehensiveness of the findings.

We have revised the Introduction to explicitly describe how this objective shaped the study design. Specifically, we clarified that NLP is positioned as

(1) a complementary tool rather than a substitute for manual TA, and

(2) an analytic approach that augments human-led coding,

and that this positioning informed both our methodological choices and our evaluation of the findings.

***Added*** (Introduction):
In the design of this study, NLP was incorporated not as a replacement for manual thematic analysis but as an exploratory, complementary method. Manual TA was first used to identify core themes, after which NLP was applied to explore additional perspectives and to generate a more structured thematic representation. This design allowed us to integrate insights from both approaches to achieve a broader understanding of caregivers’ descriptions.

In terms of evaluation, our goal was not to assess perfect concordance between manual TA and NLP. Instead, we adopted an evaluative framework that examines how the two methods jointly contribute complementary themes and illuminate different aspects of the data. This approach reflects our assumption that multiple analytic methods can reveal different layers of complex phenomena, consistent with our critical realist stance.

**2. Reliability and Transparency of NLP Outputs**

**(Section: Methods - NLP-based thematic extraction; Discussion, paragraphs 4-5)**

**While the study emphasizes “fully automated extraction,” the process of theme naming, editing, and validation remains opaque.**

**Specify which labels were generated verbatim by the model and which were rephrased or merged by researchers.**

**Describe any human-in-the-loop verification or quality control procedure.**

**Include representative raw outputs or prompt-response examples in the Supplement.**

***Response***

Thank you for this important comment. As suggested, we have added clarifications to both the main text and the Supplement to increase transparency regarding the NLP outputs and processing steps.

All theme and subcategory labels presented in this study were generated directly by the model, and no rephrasing, merging, or modification by the researchers was applied. During theme generation, we did not conduct any human-in-the-loop editing or adjustment.

The model also produced “representative response IDs” for each theme; however, the NLP system did not reliably classify individual responses into specific subcategories. In practice, many responses were incorrectly concentrated into a single subcategory, making it difficult to regard these outputs as representative examples. To prevent misinterpretation, we therefore removed the automatically generated “representative response IDs” from the NLP output table (Table 3).

To further enhance transparency, we added a brief description of the prompts used for theme generation in the Methods section and included examples of the raw model outputs in the Supplement (S2 File).

***Added*** (NLP-based thematic extraction in Methods)

All theme and subcategory labels reported in this study were generated automatically by the NLP model without any rephrasing or merging by the researchers. No human-in-the-loop editing was applied during the theme generation process.

***Added*** (Thematic extraction via NLP in Results):

Although the model produced response IDs that it associated with each theme, the accuracy of assigning individual responses to specific subcategories was insufficient; many responses were disproportionately concentrated in a single subcategory. Therefore, these automatically generated representative IDs were removed from the final presentation of the NLP results (Table 3).

To improve transparency, we provided the raw model outputs in the Supplement (S2 File).

**3. Evaluation of Concordance Between Manual and NLP Analyses**

**(Section: Discussion, first paragraph)**

**The discussion claims that both approaches identified “common themes,” but does not quantify or operationalize concordance.**

**Provide a correspondence table between manual and NLP categories or calculate thematic overlap (e.g., percentage match, semantic similarity).**

**Distinguish whether differences arise from human interpretive bias or from NLP abstraction.**

***Response***
Thank you for this comment. We agree that the degree of concordance between the manual thematic analysis and the NLP-based thematic extraction should be presented more clearly.

To address this point, we have added a correspondence table (Table 5) that explicitly maps the categories and subcategories identified by manual TA to those generated by the NLP approach. This table summarizes which themes were identified by both methods and which were unique to either approach.

We did not calculate quantitative measures of concordance, such as percentage overlap or semantic similarity scores. The correspondence between themes required contextual and conceptual interpretation rather than simple lexical matching, and we considered that reducing this process to numerical indices could be misleading.

Regarding the observed differences between the two approaches, we interpret them as reflecting differences in their analytic characteristics. Specifically, manual TA allowed researchers to generate themes informed by clinical experience and interpretive judgment, resulting in some themes that were identified only through human analysis and not by NLP. Conversely, the NLP approach identified themes that were not readily apparent through researcher-led interpretation, thereby complementing the manual analysis.

We have revised the Discussion as follows.

***Added***:

We interpret the findings by comparing the results of the manual thematic analysis and the NLP-based thematic extraction. To enhance clarity and allow readers to visually understand how themes from the two approaches correspond, we created a mapping table (Table 5) that summarizes the overlaps and divergences between subcategories. The differences observed between the two approaches reflect their distinct analytic characteristics. Manual TA enabled the generation of themes informed by researchers’ clinical experience and interpretive judgment, resulting in themes that were identified only through human analysis. Conversely, the NLP approach identified themes that were less salient from a human interpretive perspective, thereby complementing the manual analysis.

**4. Critical Examination of NLP Abstraction and Model Behavior**

**(Section: Discussion, paragraph 1; Table 2 results)**

**NLP categories such as “Parental Support Through Social and Community Networks” generalize multiple caregiver statements into high-level concepts possibly absent from the original text.**

**Discuss whether such themes represent genuine conceptual synthesis or model hallucination.**

**Acknowledge that LLMs tend to over-generalize and produce normative language.**

***Response***

Thank you for this comment.

In NLP-based thematic extraction, higher-level concepts may be generated by integrating lexical and contextual patterns that recur across multiple free-text responses. For example, the category “Parental Support Through Social and Community Networks” was generated based on recurring semantic content across responses, such as uncertainty about the future, the need for ongoing support in building parent–child relationships, and expectations for guidance from others, even when this specific wording did not appear verbatim in individual responses. Such abstraction reflects a conceptual synthesis grounded in meaning supported by the original free-text data, and in this study is interpreted not as model hallucination but as abstraction-based conceptual integration.

At the same time, as noted by the reviewer, large language models are known to have a tendency to produce overly generalized expressions or normative language when summarizing and integrating multiple narratives. Therefore, higher-level themes generated by NLP should not be interpreted as direct representations of participants’ specific accounts, but rather as products of model-driven abstraction that require cautious interpretation.

We have ***added*** the following paragraph to the Discussion:

Some of the higher-level categories generated by the NLP model include abstract concepts that do not appear verbatim in the original free-text responses. However, these categories were derived by integrating recurring semantic patterns across multiple responses, such as the need for ongoing guidance in parent–child relationships, and expectations for support from others. We therefore interpret these themes not as model hallucinations, but as instances of conceptual synthesis through abstraction. At the same time, large language models are known to have a tendency toward over-generalization and the production of normative language. Accordingly, NLP-generated higher-level themes should be interpreted cautiously, with careful attention to their faithfulness to the underlying responses [(Maynez et al. 2020)](https://sciwheel.com/work/citation?ids=16373923&pre=&suf=&sa=0).

**5. Granularity Gap Between Manual and NLP Themes**

**(Sections: Results – Manual TA vs. NLP results; Discussion, paragraph 2)**

**Manual TA yielded five fine-grained categories, while NLP produced three broad ones. The paper treats these as equivalent without clarifying analytical level.**

**Define the analytical layer used for comparison (micro vs. meso vs. macro themes).**

**If alignment occurs only at higher abstraction, explicitly say so.**

***Response***

Thank you for this comment. We revisited the analytical levels implied by the term “category” in manual thematic analysis and NLP-based thematic extraction and recognized that the two approaches operate at different levels of abstraction.

The “categories” derived from manual TA can be characterized as meso-level themes, integrating multiple concrete issues through researcher interpretation. In contrast, the “categories” generated by NLP correspond to macro-level themes, which aggregate broader content across multiple responses.

In comparison, higher concordance between the two approaches was observed at the level of subcategories, which can be positioned as micro-level themes representing specific needs and challenges. Based on this observation, we determined that direct comparison at the category level was not appropriate due to differences in analytical granularity. Instead, we focused our comparison on subcategories at the micro-level.

Accordingly, we present a mapping table (Table 5) that focuses on the correspondence between subcategories identified by manual TA and NLP. To clarify this analytical framework, we have added explicit descriptions to the Discussion section outlining the distinctions among micro-, meso-, and macro-level themes and specifying the level at which comparisons were conducted.

***Added*** (in Discussion):

For interpretive clarity, we retrospectively characterized the themes identified by manual TA and NLP in terms of their relative level of abstraction. Micro-level themes refer to concrete and specific needs directly grounded in caregivers’ descriptions, corresponding to subcategories identified by both approaches. Meso-level themes integrate multiple micro-level themes into coherent domains and largely reflect the categories generated through manual TA. In contrast, macro-level themes represent broader and more abstract constructs that aggregate multiple themes and were more characteristic of the categories generated by NLP. When comparing the two approaches, concordance was most evident at the micro-level, while differences at the meso- and macro-levels reflect the distinct analytic properties of human interpretation and computational abstraction.

**6. Ambiguity of the Study’s Core Contribution**

**(Section: Discussion)**

**It is unclear whether the main contribution is (a) developing a privacy-preserving offline NLP workflow, (b) confirming LLM–TA consistency, or (c) discovering new 22q11DS-specific needs.**

**Reframe the concluding paragraph to emphasize the primary novelty—e.g., secure LLM use in qualitative healthcare research—while treating thematic findings as an illustrative case.**

***Response***

Thank you for this comment. The central novelty of this study lies in demonstrating the feasibility of conducting NLP-assisted qualitative analysis using large language models within a fully offline, privacy-preserving environment in healthcare research. The thematic findings obtained in this study are positioned as an illustrative application of this methodological framework, rather than as the primary contribution.

Through the NLP-based analysis, we identified key themes that were consistent with those derived from manual thematic analysis, such as the need for comprehensive medical consultation systems and attention to mental health. These findings serve as concrete examples illustrating the consistency and complementarity between LLM-based analysis and manual TA.

Based on this clarification, we have restructured the concluding paragraph of the Discussion section as follows.

***Added*** (After Limitation section):

Conclusion

The primary contribution of this study is methodological. We show that NLP-assisted qualitative analysis using LLMs can be conducted within a fully offline, privacy-preserving environment in healthcare research. This workflow offers a practical approach for applying NLP to sensitive qualitative data while maintaining data security.

The thematic findings are presented as an illustrative application of this methodological approach. The NLP-based analysis identified themes—such as the need for comprehensive medical consultation systems and attention to mental health—that were consistent with those derived from manual TA. Together, these results suggest that NLP would complement, rather than replace, human-led qualitative analysis in medical research.

**7. Incomplete Discussion of NLP Methodological Limitations**

**(Section: Limitations)**

**The Limitations section omits discussion of NLP-specific weaknesses: cultural and linguistic bias, prompt dependency, loss of contextual nuance, and difficulty attributing quotes to individuals.**

**Add explicit acknowledgment that LLMs may misinterpret idiomatic Japanese caregiver expressions or generate synthetic phrasing.**

**State that AI-based extraction is suitable for overview analysis but not for capturing emotional or contextual subtleties.**

***Response***

Thank you for this comment. As noted by the reviewer, the NLP-based thematic extraction used in this study has several inherent limitations. In light of these limitations, our intention was not to interpret the NLP-derived results in isolation, but rather to use them in conjunction with manual thematic analysis in order to compensate for the limitations of NLP while obtaining an overall understanding of the data. Accordingly, we have explicitly added a discussion of NLP-specific methodological weaknesses and related interpretive considerations to the Limitations section.

***Added*** (in Limitations):

Sixth, LLMs may incorporate cultural and linguistic biases derived from their training data and model design (Bender et al. 2021). In particular, free-text responses written by Japanese caregivers often include idiomatic expressions and implicit meanings, which LLMs may misinterpret or over-generalize during abstraction. Seventh, NLP-based thematic extraction is inherently dependent on prompt design, and even minor variations in prompts may influence the resulting outputs. In addition, when summarizing and integrating multiple narratives, LLMs may generate synthetic or normative phrasing that does not appear verbatim in the original text, raising concerns about faithfulness to the source data (Maynez et al. 2020). Eighth, while NLP-based analysis is useful for obtaining an overview of general trends and shared patterns across large volumes of qualitative data, it is less suited to capturing emotional intensity and subtle contextual nuances embedded in individual narratives (Grimmer and Stewart 2013).

**Minor Points**

**1. Reference Contextualization**

**(Introduction & Discussion)**

**When citing prior ChatGPT-based TA studies (refs 10, 11, 22), specify how your approach differs - e.g., local processing, open-source model, or ethics compliance. A concise comparison table would improve clarity.**

***Added*** (Discussion):

A key methodological contribution of this study lies in demonstrating the feasibility of conducting NLP-assisted qualitative analysis within a fully offline, privacy-preserving environment. Previous applications of large language models (LLMs) for thematic analysis relied on cloud-based systems (22), which posed challenges when handling sensitive personal narratives. In this study, we developed an analysis workflow that employs an open-source LLM executed entirely on local hardware, enabling secure processing of confidential qualitative data (Table 4).

**2. Positionality Statement Integration**

**(Discussion, paragraph 1 middle)**

**The authors mention “researchers’ positionality shaped by clinical experience,” but this appears abruptly. Move this to Limitations or Methods and explicitly state how positionality informed interpretation.**

***Response***

Thank you for this comment. In this study, the researchers’ positionality, shaped by their clinical experience, may have influenced the interpretation and integration of themes in the manual thematic analysis. We agree that this is an important factor in interpreting the findings. Accordingly, we have added an explicit description to the Methods section detailing the researchers’ clinical backgrounds and how their positionality may have informed the analytical and interpretive processes. This revision clarifies the potential influence of researcher positionality on the analysis and helps readers better understand its role in shaping the findings.

***Added*** (Methods):

Reflexivity was maintained throughout the analysis. The two coders brought distinct forms of expertise and positionality: the first author is a psychiatrist working in a specialized outpatient clinic for individuals with 22q11.2 deletion syndrome and is a beginner in thematic analysis, whereas the second author is a clinical psychologist with substantial qualitative research experience and close engagement with participants through recruitment, communication, and follow-up. Analytic memos were shared to reflect on how these backgrounds might influence interpretations, and potential biases were discussed and adjusted during the refinement of categories and themes. This iterative process ensured that interpretations did not become overly shaped by a single researcher’s standpoint.

**3. Participant Representation and Bias**

**(Limitations)**

**The sampling bias description is appropriate but could specify which demographic or clinical characteristics are overrepresented. If known, briefly compare responders and non-responders.**

***Response***

Thank you for this helpful comment. To clarify potential sampling bias, we compared the characteristics of respondents and non-respondents to the free-text question on medical needs. We added the demographic characteristics of respondents to Table 1 and the clinical characteristics of non-respondents to Supplementary Table S1, thereby making explicit which groups may be overrepresented in the analytic sample.

The comparison showed no substantial differences in mean age or sex distribution between respondents (n = 65) and non-respondents (n = 60). In contrast, respondents had a greater variety of physical comorbidities, with slightly higher prevalence of conditions such as congenital heart disease, immune system disorders, and endocrine disorders. In addition, the prevalence of psychiatric disorders was approximately twice as high among respondents compared with non-respondents (36.9% vs. 18.3%).

These findings suggest that families facing greater medical and psychiatric challenges may have been more likely to provide free-text responses. We therefore revised the Limitations section to explicitly note the possibility that respondents represent a subgroup with more complex comorbidities, and to discuss the implications of this potential bias for interpretation of the findings.

***Added*** (Limitation):

There also may be sampling bias between respondents (Table 1) and non-respondents (Table S1) to the free-text question on medical needs. Although no substantial differences were observed in mean age or sex distribution, respondents exhibited a greater variety of physical comorbidities, with slightly higher prevalence of conditions such as congenital heart disease, immune system disorders, and endocrine disorders. In addition, the prevalence of psychiatric disorders among respondents was approximately twice that observed among non-respondents (36.9% vs. 18.3%). These patterns suggest that families experiencing more complex medical and psychiatric challenges may have been more likely to provide free-text responses, and thus the results of this study may disproportionately reflect the perspectives of individuals with more complex healthcare needs.

**4. Translation and Language Transparency**

**(Methods – data processing; Results tables)**

**Since qualitative data were originally in Japanese, describe translation workflow (e.g., who translated, whether back-translation was used). This is vital for replicability and cross-cultural interpretation.**

Thank you for this comment. The themes generated in this study and the quoted free-text responses were translated from Japanese into English by the first author. Although back-translation was not performed, the accuracy and appropriateness of the translations were reviewed by multiple healthcare professionals with extensive clinical experience in caring for individuals with 22q11.2 deletion syndrome. This process ensured that the medical context and implied meanings were appropriately preserved. Based on your suggestion, we have added a description of the translation workflow to the relevant section of the Results.

***Added*** (in Results):

The themes and quoted free-text responses presented in this study were translated from Japanese into English by the first author. Although back-translation was not performed, the translations were reviewed by multiple healthcare professionals with clinical experience in the care of individuals with 22q11.2 DS to ensure accuracy and preservation of medical context and implied meanings.

**5. Terminological Consistency**

**(Throughout Results & Discussion)**

**Unify expressions such as “comprehensive support,” “comprehensive improvement,” and “integrated care.” Consistent terminology will make the comparison between manual and NLP themes clearer.**

**Check for other inconsistent expressions throughout the manuscript as well.**

***Response***

Thank you for this comment. We agree that the terminology used in the Results and Discussion sections was not sufficiently consistent. In response to the reviewer's suggestion, we reviewed and standardized the terminology throughout the manuscript. Specifically, we now use the term “comprehensive support” consistently to refer to concepts that encompass medical, welfare, and psychological support in an integrated manner. Expressions such as “integrated care” and “comprehensive improvement” were revised to “comprehensive support” where appropriate, or retained only with additional clarification when required by the context. In addition, because some variability arose during the translation of theme labels from Japanese to English, we have similarly standardized the English expressions of theme names. These revisions have been implemented as described below.

Abstract

***Deleted***: comprehensive improvement of

***Added***: comprehensive support across

Results (Manual Thematic Analysis)

***Deleted***: comprehensive consultation services

***Added***: comprehensive support services

***Deleted***: integrated medical care

***Added***: comprehensive medical support

Table 2

***Deleted***: a comprehensive consultation service

***Added***: comprehensive support services

***Deleted***: integrated medical care

***Added***: comprehensive medical support

Results (Thematic extraction via NLP)

***Deleted***: Comprehensive Improvement of

***Added***: Comprehensive Support Across

***Deleted***: integrated care

***Added***: comprehensive support

Table 3

***Deleted***: Comprehensive Improvement of

***Added***: Comprehensive Support Across

***Deleted***: integrated clinical care

***Added***: comprehensive medical care

**6. Minor Editorial Corrections**

**Correct typographical error “22q12DS” -> “22q11DS” in Limitations.**

**Improve table formatting so Tables 1 and 2 share identical columns (“Theme / Subtheme / Illustrative Quote / Participant ID”).**

**If “22q12DS” actually exists as a distinct condition, briefly explain it to avoid confusion.**

***Response***

Thank you for this comment. The term “22q12DS” in the Limitations section was a typographical error and has been corrected to “22q11DS.”

Regarding the table formatting, Table 1 (Table 2 in the current manuscript) and Table 2 (Table 3 in the current manuscript) present results derived from different analytical approaches, and therefore their column structures were not unified. The table based on manual thematic analysis includes illustrative quotes to facilitate interpretation of the themes. In contrast, for the NLP-based thematic extraction, illustrative quotes were omitted to avoid potential misinterpretation, as the NLP approach had limited ability to accurately assign individual responses to specific themes.

***Deleted***: 22q12DS

***Added***: 22q11DS

**Concluding Assessment**

**The manuscript addresses an important intersection of qualitative health research and natural language processing. Its strengths lie in ethical rigor and methodological innovation through offline LLM deployment. Nonetheless, the argumentation would benefit from tighter alignment between aims, methods, and interpretations.**

**Specifically, the paper should:**

**1. Clarify the analytic goal of combining TA and NLP,**

**2. Quantitatively or systematically evaluate thematic concordance,**

**3. Disclose the extent of human intervention in AI outputs, and**

**4. Critically reflect on the abstraction tendencies and cultural limitations of LLMs.**

**Addressing these points will greatly enhance methodological transparency, theoretical coherence, and the overall scientific contribution of the study.**

***Response***

Thank you for this comprehensive and constructive assessment. In response to these points, we have revised the manuscript to improve alignment between the study aims, methods, and interpretations. We believe that these revisions substantially strengthen the methodological transparency, theoretical coherence, and overall contribution of the study.
